# Musculoskeletal Findings Following Pre-Participation Screening in Young Athletes: A Retrospective Observational Study

**DOI:** 10.3390/jfmk11040413

**Published:** 2026-10-08

**Authors:** Marco Vecchiato, Stefano Palermi, Luisa Ancona, Giulio Barbaro, Giampietro Brontesi, Rosario Conte, Fulvio De Luca, Giulia Foccardi, Luigi Gerbino, Mattia Grassi, Vincenzo Pandolfi, Giulia Quinto, Carlo Sutto, Stefano Varponi, Stefano Bargellesi, Erica Brugin

**Affiliations:** 1 Sports Medicine and Cardiovascular Rehabilitation Unit, Unità Locale Socio Sanitaria (ULSS) 3 Serenissima, Noale, 30033 Venice, Italy; 2 Sports and Exercise Medicine Division, Department of Medicine, University of Padova, 35128 Padova, Italy; 3 Department of Theoretical and Applied Sciences, eCampus University, 22060 Novedrate, Italy; 4 Department of Medicine and Surgery, UniCamillus-Saint Camillus International University of Health Sciences, 00187 Rome, Italy; 5 Rehabilitation Medicine Department, Unità Locale Socio Sanitaria (ULSS) 3 Serenissima, Noale, 30033 Venice, Italy

**Keywords:** pre-participation screening, youth athletes, musculoskeletal screening, sports medicine, adolescent, injury prevention

## Abstract

**Background**: Pre-participation screening (PPS) is widely used to evaluate athletes before sport participation, traditionally focusing on cardiovascular risk prevention. However, musculoskeletal assessment performed during PPS may also allow early identification of biomechanical and structural abnormalities that can affect sport participation during growth. **Objectives**: This study aimed to describe the prevalence, clinical characteristics, and management pathways of musculoskeletal alterations detected during PPS in young athletes, with particular focus on referral patterns to pediatric Physical Medicine and Rehabilitation (PM&R) specialists. **Methods**: This retrospective observational study included athletes aged ≤15 years who underwent PPS between January 2022 and December 2023 at the Sports Medicine Unit of Noale (ULSS 3 Serenissima, Venice, Italy). Musculoskeletal screening was systematically performed during PPS. Athletes presenting newly detected abnormalities were either managed conservatively by the sports physician or referred for PM&R evaluation. Clinical indications for referral, imaging investigations, diagnoses, and treatment strategies were analyzed. **Results**: A total of 707 athletes underwent PPS during the study period. After applying exclusion criteria, 635 athletes were eligible for analysis. Musculoskeletal alterations were identified in 141 athletes (22.2%). Among them, 54 athletes were referred for PM&R evaluation and 40 completed the consultation. No statistically significant differences were observed between referred and non-referred athletes. The most frequent reasons for referral were suspected scoliosis (*n* = 22), foot alignment disorders *n* = 18), thoracic hyperkyphosis (*n* = 5), and lower limb length discrepancy (*n* = 4). Specialist evaluation resulted in conservative management in most cases, including corrective exercise programs and orthotic interventions, while one athlete required surgical treatment for congenital scoliosis. **Conclusions**: Musculoskeletal screening during PPS identified a broad spectrum of findings in young athletes, including clinically relevant abnormalities requiring specialist evaluation and management. These findings suggest that musculoskeletal assessment may provide an opportunity for early clinical identification within PPS; however, prospective studies are needed to evaluate diagnostic accuracy, long-term clinical benefit, injury prevention, and cost-effectiveness before routine implementation can be recommended.

## 1. Introduction

Pre-participation screening (PPS) is a cornerstone of sports medicine and is primarily aimed at assessing eligibility for sport participation and identifying conditions that may increase the risk of adverse events [1]. Although traditionally focused on cardiovascular screening [2], PPS also provides an opportunity to assess other clinically relevant domains, including the musculoskeletal system [3]. In young athletes, musculoskeletal assessment may be particularly relevant because growth, skeletal maturation, and sport-specific loading can contribute to functional and structural abnormalities that may influence sport participation [4,5].

In pediatric and adolescent populations, musculoskeletal complaints are common and may reflect transient functional adaptations, growth-related conditions, or early manifestations of structural pathology [6]. Conditions such as adolescent idiopathic scoliosis, flexible flatfoot, limb length discrepancy, and apophyseal traction disorders have been associated with altered movement patterns and increased injury susceptibility [7,8,9]. Moreover, intensive sport participation during skeletal maturation may exacerbate underlying biomechanical asymmetries or contribute to overload syndromes. Growing evidence suggests that early identification of postural alterations, spinal deformities, and biomechanical imbalances may support timely clinical management and targeted interventions during critical growth phases [10,11].

The recent literature has emphasized the need for multidisciplinary approaches in youth sports medicine, integrating screening, functional assessment, and, when indicated, targeted rehabilitation interventions or orthotic management [12,13]. Specialist referral is clinically relevant because it links abnormal screening findings to diagnostic confirmation and subsequent management. Although previous pediatric PPS studies have reported musculoskeletal abnormalities and specialist referrals, evidence on referral yield and downstream diagnostic and management pathways remains limited [3,8,9,10].

Important knowledge gaps therefore remain regarding the prevalence of clinically relevant musculoskeletal findings detected during PPS, the characteristics of athletes requiring specialist referral, the diagnostic yield of specialist evaluation, and subsequent management pathways [5,14]. Better characterization of these aspects may help clarify the clinical utility of musculoskeletal assessment within PPS and inform the development of more standardized referral pathways.

Accordingly, the aim of this study was to describe the prevalence and clinical characteristics of musculoskeletal findings detected during PPS in young athletes and to characterize subsequent referral patterns, specialist diagnostic findings, and management strategies.

## 2. Materials and Methods

### 2.1. Study Design

This was a retrospective observational study conducted on young athletes evaluated at the Sports Medicine Unit of Noale, ULSS 3 Serenissima (Venice, Italy). The study included athletes undergoing annual PPS for sports eligibility between 1 January 2022 and 31 December 2023, according to the Italian national regulations governing competitive sports participation. For athletes with more than one PPS visit during the study period, only the first eligible examination was considered as the index visit so that each athlete contributed a single observation to the analysis. The clinical data analyzed in the present study were generated and recorded during routine PPS visits performed between 2022 and 2023. Their retrospective use for research purposes, analysis, and dissemination was undertaken only after approval by the Territorial Ethics Committee of the Central-East Veneto Area on 4 June 2026 (protocol code: NO LIMIT; 6657/U3/26). The approved protocol covered the retrospective analysis of sports medicine evaluations performed at ULSS 3 Serenissima over a substantially broader historical period, including the 2022–2023 period considered in the present study. 

In Italy, PPS represents a structured medical evaluation aimed at determining sports eligibility and preventing sport-related adverse events [15,16]. The standard protocol includes a comprehensive clinical and functional assessment, performed annually for competitive athletes [17]. During PPS, athletes underwent a detailed personal and family medical history assessment, a comprehensive general physical examination, resting 12-lead electrocardiography, spirometry, urinalysis, visual assessment including visual acuity, hearing evaluation, and a submaximal exercise test with continuous ECG monitoring [18]. Blood pressure was measured at rest, at peak exercise, and during the recovery phase.

### 2.2. Study Population

All consecutive athletes aged ≤15 years undergoing PPS during the study period were considered eligible. Athletes were excluded if they had a previously diagnosed musculoskeletal disorder, a neurological disease affecting motor function, or a known orthopedic condition already under specialist management. Sporting disciplines were classified according to the predominant exercise component (skill, power, mixed, and endurance), in accordance with the 2020 ESC Guidelines on sports cardiology and the 2023 COCIS recommendations [19,20].

### 2.3. Musculoskeletal Evaluation

In routine Italian PPS, musculoskeletal assessment is generally incorporated into the clinical physical examination but is not based on a universally standardized structured protocol. During the study period, a systematic structured musculoskeletal assessment was incorporated into routine clinical PPS practice and documented in the athletes’ medical records. All athletes underwent a systematic structured musculoskeletal assessment, which was performed by the same sports medicine physician with extensive experience in musculoskeletal assessment of young athletes, in order to ensure consistency of the screening procedure and minimize inter-examiner variability. The examination followed a predefined sequence including postural inspection, spinal assessment, lower limb symmetry and alignment, foot morphology and gait observation, and selected joint- and apophysis-specific clinical tests. Qualitative postural findings, including shoulder or scapular asymmetry, pelvic tilt, lower-limb axial alignment, and gait deviations, were recorded descriptively on clinical inspection. The duration of the musculoskeletal screening was timed in the first 30 consecutive athletes undergoing the study-specific assessment.

#### 2.3.1. Postural Inspection

Postural assessment was performed with the athlete in a relaxed standing position and consisted of a systematic visual inspection of body alignment in both frontal and sagittal planes. Particular attention was paid to the alignment of the head and shoulders, scapular symmetry, thoracic and lumbar curvature, pelvic alignment, and lower limb axis. The examination aimed to detect possible postural asymmetries such as scapular dyskinesis, pelvic tilt, or abnormal weight distribution between the lower limbs. These observations allowed the clinician to identify functional postural adaptations that might reflect underlying biomechanical imbalances or compensatory mechanisms.

#### 2.3.2. Spinal Alignment Assessment

Spinal alignment was assessed through clinical inspection combined with functional testing. The evaluation included observation of sagittal plane curvatures, particularly thoracic kyphosis and lumbar lordosis, as well as inspection of possible coronal plane deviations suggestive of scoliotic posture or structural scoliosis. The Adams forward bending test was systematically performed to detect trunk asymmetry or a rib hump suggestive of vertebral rotation. When asymmetry was observed, the angle of trunk rotation was measured using a scoliometer, positioned perpendicular to the longitudinal axis of the spine at the level of maximal trunk prominence.

#### 2.3.3. Lower Limb Symmetry Evaluation

Assessment of the lower limbs focused on identifying asymmetries or alignment abnormalities potentially affecting biomechanical function during sports activities. Lower limb length was initially assessed with the athlete in the supine position to identify true anatomical discrepancies. When clinically indicated, the lengths of individual lower limb segments were also measured to better characterize the origin of the discrepancy. The examination was subsequently repeated in the standing position to assess pelvic leveling, lower limb alignment, and the functional consequences of any detected discrepancy under weight-bearing conditions. Particular attention was also paid to the knee axis, including the presence of valgus or varus alignment. This combined assessment allowed differentiation between true limb length discrepancy and apparent asymmetry potentially related to pelvic malalignment or altered foot support. Minor asymmetries without clinically relevant functional consequences were considered physiological and managed through routine monitoring, whereas discrepancies associated with pelvic imbalance or clinically relevant biomechanical alterations were considered significant and prompted specialist referral.

#### 2.3.4. Foot Morphology and Gait Observation

Foot morphology was assessed through clinical examination and static observation using a conventional podoscope. Clinical inspection focused on morphological features such as flexible flatfoot, cavus foot, and hindfoot valgus or varus. The podoscope was used during static weight-bearing to visually assess the plantar footprint and medial longitudinal arch. Gait was assessed separately by visual observation during walking. No quantitative plantar-pressure measurements or dynamic baropodometric analyses were performed.

#### 2.3.5. Routine and Symptom-Guided Clinical Tests

As part of the systematic musculoskeletal examination, all athletes underwent a series of additional clinical tests. These included bilateral palpation of the tibial tuberosities and calcaneal apophyses, anterior drawer testing of the knee, assessment of patellar mobility, and clinical evaluation of ankle stability.

Additional region-specific orthopedic tests were performed only when symptoms or abnormal findings identified during the initial examination warranted further assessment.

### 2.4. Referral

Referral to pediatric Physical Medicine and Rehabilitation (PM&R) specialists was based on predefined objective criteria where available and on predefined clinical criteria requiring physician judgment for findings not amenable to a single numerical threshold. Objective referral criteria included (i) a visible rib hump on the Adams forward bending test corresponding to an angle of trunk rotation ≥5° on scoliometer assessment, and (ii) a true lower limb length discrepancy >1 cm, confirmed clinically in the supine position and associated with pelvic obliquity or functional asymmetry during standing.

For findings without a predefined numerical threshold within the study protocol, referral was based on specific clinical characteristics. Foot abnormalities prompted referral when associated with pain, exercise-related fatigue, marked asymmetry, poor correctability, or ankle instability. Thoracic hyperkyphosis prompted referral when clinically pronounced, poorly correctable, or associated with dorsal pain. Additional indications included persistent or recurrent spinal or limb pain, focal bony tenderness, prominent structural deformity, or abnormal knee or ankle stability findings. Mild, flexible, and asymptomatic findings without these characteristics were managed directly by the sports physician through education, behavioral or postural recommendations, as well as clinical follow-up.

### 2.5. Outcomes

The primary outcomes were the prevalence of newly detected musculoskeletal findings among eligible athletes and the proportion of athletes referred for pediatric PM&R evaluation. Secondary outcomes included referral indications, completion of specialist evaluation, specialist diagnoses, imaging investigations, and subsequent management among athletes who attended the PM&R consultation. Exploratory analyses assessed demographic, anthropometric, and sport-related characteristics according to musculoskeletal screening and referral status, where data availability permitted.

### 2.6. Statistical Analysis

Descriptive statistics were used to summarize the data. The distribution of continuous variables was assessed using the Shapiro–Wilk test. Continuous variables were reported as mean ± standard deviation when normally distributed and as median and interquartile range (IQR) when non-normally distributed. Categorical variables were expressed as absolute numbers and percentages. The prevalence of PM&R referrals and the distribution of diagnostic categories were calculated for the entire cohort. For the main prevalence estimates, 95% confidence intervals (CIs) were calculated using the Wilson score method. Athletes who did not complete the recommended PM&R consultation remained included in the screening and referral analyses. Downstream diagnostic, imaging, and management outcomes were considered unavailable for these athletes and were analyzed only among those who completed the specialist evaluation. No imputation was performed. All statistical analyses were performed using SPSS version 26.0 software.

## 3. Results

### 3.1. Study Cohort and Referral Pathway

**Figure 1:**
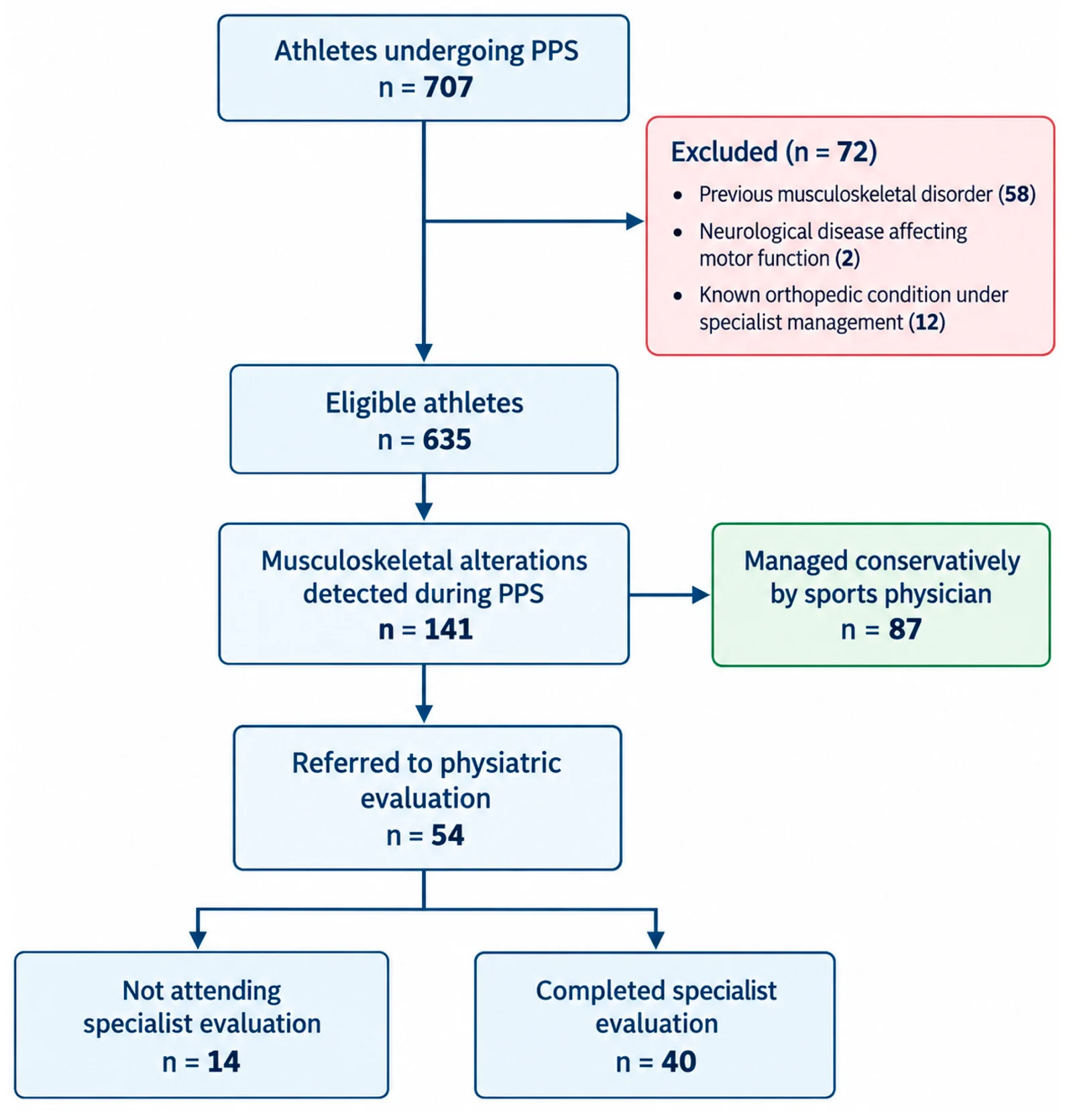
Flowchart of athlete selection and referral pathway following pre-participation screening.

During the study period, 707 athletes aged ≤15 years underwent annual PPS. The median screening duration was 2 min and 10 s (range, 1 min 50 s to 2 min 55 s). Seventy-two athletes (10.2%) were excluded: 58 had a previously diagnosed musculoskeletal disorder, 2 had a neurological disease affecting motor function, and 12 had a known orthopedic condition already under specialist management. The remaining 635 athletes constituted the eligible cohort. Musculoskeletal alterations were identified in 141 of 635 athletes (22.2%; 95% CI 19.1–25.6). Among these, 54 of 141 athletes were referred for pediatric PM&R evaluation (38.3%; 95% CI 30.7–46.5), corresponding to 8.5% of the eligible cohort (95% CI 6.6–10.9). Forty of the 54 referred athletes completed the specialist consultation (74.1%; 95% CI 61.1–83.9). The 87 athletes who did not require specialist referral mainly presented with low-grade postural asymmetries, mild scoliotic attitudes or small trunk asymmetries below the referral threshold, mild hyperkyphotic posture, early traction-apophysis tenderness, and mild flexible flatfoot. These findings were managed conservatively with education, postural advice, stretching or mobility exercises, training-load modification when appropriate, and periodic clinical reassessment. Characteristics of athletes with musculoskeletal findings according to referral status are summarized in Table 1. There were no statistically significant differences between referred and non-referred athletes in demographic, anthropometric, or sport-related characteristics (all *p* > 0.05). The study population and referral pathway are shown in Figure 1.

### 3.2. Reasons for Specialist Referral

Referral indications were available for all 54 athletes referred for PM&R evaluation, irrespective of subsequent attendance at the specialist consultation (Table 2). The reasons for referral to the PM&R specialist were grouped into major diagnostic categories.

The most frequent indication for referral was suspected spinal deformity, identified through the presence of trunk asymmetry or rib hump during clinical examination. A total of 22 athletes were referred for the presence of a hump deformity suggestive of scoliosis. Foot abnormalities represented the second most frequent referral category, involving 18 athletes overall: 12 with flexible flatfoot/hindfoot valgus and 6 with cavus foot morphology. Five athletes were referred for dorsal pain associated with thoracic hyperkyphosis, while four were referred because of suspected lower limb length discrepancy. Seven athletes were referred for other musculoskeletal complaints, including flat-back posture with dorsal pain, chronic low back pain, lumbar pain with tenderness over the sacral region, prominent bilateral hallux valgus, and heel pain. Importantly, the vast majority of athletes referred for specialist evaluation were asymptomatic at the time of PPS (47/54, 87%), and the musculoskeletal abnormalities were detected exclusively through the systematic clinical examination performed by the sports physician.

### 3.3. Outcomes of Specialist Evaluation

Among athletes who completed specialist PM&R evaluation, the diagnostic findings, imaging investigations, and management strategies are summarized in Table 3. For referral categories with a directly corresponding specialist diagnosis, the suspected abnormality was confirmed in all evaluated cases: scoliosis in 16/16 athletes, foot morphology abnormalities in 14/14, thoracic hyperkyphosis in 3/3, and clinically significant lower limb length discrepancy in 3/3.

All 16 athletes evaluated for suspected scoliosis underwent spinal radiography, leading to the diagnosis of idiopathic scoliosis in 15 cases and congenital scoliosis due to a lumbar hemivertebra in one case; the latter also underwent MRI and required orthopedic evaluation and surgical treatment. The remaining scoliosis cases were managed with bracing and/or therapeutic exercise or clinical follow-up.

Among athletes evaluated for foot abnormalities, nine were diagnosed with flexible flatfoot with hindfoot valgus and five with cavus foot; management consisted mainly of plantar orthoses and/or therapeutic exercise. Thoracic hyperkyphosis was confirmed in three athletes and managed conservatively with exercise, follow-up, and, in one case, bracing. Clinically significant lower limb length discrepancy was confirmed in three athletes, all of whom received a unilateral plantar lift.

The remaining specialist diagnoses included calcaneal apophysitis, flat-back syndrome, lumbar disk protrusions, L5 spondylolysis with minimal L5–S1 anterolisthesis, and bilateral hallux valgus. These conditions were managed predominantly through conservative treatment, rehabilitation, activity modification, or orthotic treatment.

## 4. Discussion

In this retrospective cohort of 635 eligible young athletes undergoing PPS, musculoskeletal findings were identified in 22.2% of athletes. Overall, 8.5% of the eligible cohort and 38.3% of athletes with musculoskeletal findings were referred for specialist PM&R evaluation. Among referred athletes, 74.1% completed the specialist assessment. The detected abnormalities covered a broad clinical spectrum, ranging from minor abnormalities managed directly by the sports physician to conditions requiring specialist assessment, imaging, orthotic or rehabilitative management and, in one case, surgical treatment.

These observations emphasize the role of the sports physician not only in determining eligibility for sport participation but also in acting as a first-line clinician for the early recognition of musculoskeletal disorders during growth [21,22].

Our results can be directly compared with those of Palermi et al., who reported abnormal pediatric Gait, Arms, Legs, Spine (pGALS) findings in approximately 40% of 654 apparently healthy children undergoing sports pre-participation evaluation [10]. The lower prevalence observed in our cohort (22.2%) may partly reflect methodological differences. Palermi et al. used the broader pGALS screening tool, which systematically assesses gait, arms, legs, and spine and may identify a wider spectrum of minor abnormalities, whereas the present study applied a focused musculoskeletal assessment within PPS, with particular attention to findings potentially relevant to clinical management and specialist referral. Differences in age distribution may also have contributed, as the cohort studied by Palermi et al. was younger (mean age 8.9 years). Importantly, their study emphasized that abnormal screening findings require interpretation within the broader clinical context. More broadly, the recent literature on young athletes indicates that approximately 3–14% may require additional evaluation following PPS [3]. Our specialist referral rate of 8.5% of the eligible cohort falls within this range. However, direct comparisons should be interpreted cautiously because previous estimates encompass different clinical indications for further evaluation and were derived from heterogeneous populations, screening protocols, and referral criteria.

Notably, most athletes referred for specialist evaluation were asymptomatic at the time of PPS. This finding indicates that clinically relevant musculoskeletal findings may be detected during examination even in the absence of reported symptoms. Indeed, a key aspect emerging from this study is the potential of PPS to function as a preventive health strategy [9,13]. In growing athletes, musculoskeletal structures are still undergoing maturation, and repeated sport-specific loading may exacerbate underlying biomechanical imbalances if they remain unrecognized [23]. However, the present study cannot determine whether these abnormalities would otherwise have remained unidentified, subsequently become symptomatic, or progressed over time. Similarly, whether their identification and management translate into improved symptoms, reduced injury incidence, or better long-term sport participation cannot be determined from the present data.

Among the conditions detected through screening, suspected scoliosis represented the most frequent indication for specialist referral. Adolescent idiopathic scoliosis is one of the most common spinal deformities during growth, with a reported prevalence of approximately 2–3% in the general pediatric population [7,24]. Early detection is considered crucial because timely intervention, particularly with bracing and targeted rehabilitation, can prevent progression of the curvature and reduce the likelihood of surgical treatment [25,26]. The present study illustrates how PPS can facilitate early identification of spinal asymmetries, enabling prompt diagnostic imaging and specialist evaluation [27,28]. In one case, screening led to the detection of severe congenital scoliosis caused by a hemivertebra, ultimately requiring surgical correction. This finding underscores the potential clinical impact of systematic spinal evaluation during sports medical visits.

Foot morphology abnormalities represented another frequent reason for specialist evaluation. Alterations such as flexible flatfoot or cavus foot are common during childhood and adolescence and may influence lower limb biomechanics and load distribution [8,29]. Although plantar orthoses were commonly prescribed in our cohort, particularly for flexible flatfoot with hindfoot valgus, these prescriptions represent observed clinical management rather than evidence of treatment effectiveness. The management of asymptomatic pediatric flexible flatfoot remains controversial [30,31], and current evidence does not support routine orthotic treatment of painless flexible flatfoot [32,33]. Accordingly, our findings should not be interpreted as supporting orthotic treatment of asymptomatic foot morphology abnormalities per se.

Spinal pain associated with thoracic hyperkyphosis represented another relevant clinical scenario observed during screening. Postural alterations of the thoracic spine are relatively common in adolescents and may be related to rapid growth, muscular imbalance, or prolonged sedentary behavior [26,34,35]. In young athletes, these alterations may also reflect sport-specific adaptations or training-related overload [9]. Conservative management based on corrective exercise programs and postural rehabilitation is widely recommended and was adopted in all cases in our cohort. Early intervention is particularly important during adolescence, when spinal curvature remains modifiable.

Lower limb length discrepancy was also identified in a small number of athletes. Although mild discrepancies are common and often asymptomatic, larger differences may affect pelvic alignment and gait biomechanics [36,37]. In the present study, clinically relevant discrepancies were managed through the prescription of plantar lifts aimed at restoring biomechanical balance [38,39]. Early identification of these alterations may contribute to preventing compensatory mechanisms that could predispose athletes to overuse injuries [40].

An important distinction is therefore required between the presence of a musculoskeletal finding and the presence of a condition requiring specialist evaluation. In our cohort, 87 athletes with screening findings were managed without referral because the abnormalities were considered minor, flexible, or of limited clinical significance. This distinction is particularly important in asymptomatic children and adolescents, in whom anatomical and postural variants are common. Broadening musculoskeletal screening without clearly defined thresholds could increase unnecessary referrals, investigations, parental concern, and medicalization of benign variants. Referral criteria should therefore prioritize findings with sufficient clinical relevance while avoiding the assumption that every screening abnormality represents disease [9].

An important practical aspect is the limited additional time required for the musculoskeletal assessment. In the first 30 consecutive athletes in whom screening duration was measured, the median examination time was 2 min and 10 s (range, 1 min 50 s to 2 min 55 s), suggesting that the protocol can be completed with a modest additional time burden within PPS. However, resource utilization and downstream healthcare costs were not systematically assessed. Therefore, the present study cannot establish the economic sustainability or cost-effectiveness of this approach. The limited screening time, together with the identification of clinically significant conditions in selected athletes, supports further investigation of its potential clinical and economic implications.

Future research should prospectively validate musculoskeletal PPS protocols in larger multicenter cohorts and establish standardized examination and referral criteria. Studies should assess diagnostic accuracy against appropriate reference standards, inter- and intra-rater reliability, and the consequences of both positive and negative screening findings. Longitudinal follow-up is also needed to determine whether detected abnormalities are associated with subsequent symptoms, injury, functional limitation, or changes in sport participation and whether interventions triggered by screening improve these outcomes. Finally, formal analyses of resource utilization and cost-effectiveness will be required before systematic implementation can be recommended.

## 5. Study Limitations

Several limitations should be considered when interpreting these findings. First, the retrospective observational design and use of routinely collected clinical data limited the availability and uniformity of some individual-level variables. In particular, a fully characterized comparison group was not available because detailed demographic, anthropometric, and sport-related covariates were not systematically available for athletes with a normal musculoskeletal screening. Consequently, determinants of abnormal screening findings could not be reliably evaluated. The single-center setting may also limit generalizability to healthcare systems using different PPS or referral models. Second, specialist follow-up was incomplete. Athletes who did not attend the recommended PM&R evaluation may have differed from attendees in clinical severity or other relevant characteristics, introducing potential attrition bias. Consequently, the diagnostic and management outcomes observed among athletes completing specialist evaluation may not be fully representative of all referred athletes. Third, the study was not designed as a diagnostic-accuracy investigation. No systematic reference-standard assessment was performed in athletes who were not referred, precluding estimation of sensitivity, specificity, false-positive rates, or false-negative rates. Although all musculoskeletal examinations were performed by the same experienced sports medicine physician, thereby minimizing inter-examiner variability, formal intra-rater reliability was not assessed, and some clinical findings and referral decisions remained partly dependent on examiner judgment.

Finally, longitudinal data on symptom progression, subsequent musculoskeletal injury, functional outcomes, and sport participation were not available. This study therefore cannot determine whether abnormalities detected during PPS are associated with future injury risk or whether the treatments prescribed following specialist evaluation improve subsequent clinical outcomes. Resource utilization and healthcare costs were also not formally evaluated.

## 6. Conclusions

In this retrospective cohort of young athletes undergoing PPS, musculoskeletal findings were identified in approximately one in five athletes, while a smaller proportion required specialist PM&R evaluation. Specialist assessment identified a range of spinal, foot, and lower-limb abnormalities, which were predominantly managed conservatively, although one clinically significant congenital spinal condition required surgical treatment. Musculoskeletal assessment during PPS may therefore provide an opportunity for early clinical identification and appropriate referral of selected abnormalities. However, the present findings do not establish diagnostic accuracy, injury-prevention benefit, treatment effectiveness, or cost-effectiveness, and prospective multicenter validation is required before systematic implementation can be recommended.

## Figures and Tables

**Table 1:** Characteristics of athletes with musculoskeletal alterations detected during PPS.

Variable	All Athletes with MSK Findings (*n* = 141)	Not Referred to PM&R (*n* = 87)	Referred to PM&R (*n* = 54)
Age, years	12.9 ± 1.6	12.8 ± 1.7	13.0 ± 1.5
Sex (male), *n* (%)	82 (58%)	52 (59.8%)	30 (55.6%)
Height, cm	156.2 ± 10.4	155.7 ± 10.7	157.0 ± 10.0
Weight, kg	48.1 ± 11.2	47.5 ± 11.5	49.0 ± 10.8
Body Mass Index (BMI), kg/m^2^	19.5 ± 2.6	19.4 ± 2.7	19.6 ± 2.5
BMI percentile	53 (IQR 38–69)	52 (IQR 36–67)	56 (IQR 40–72)
Age at sport initiation, years	8.1 ± 2.1	8.2 ± 2.2	8.0 ± 2.0
Years of sport participation	4.3 ± 2.2	4.1 ± 2.3	4.6 ± 2.1
Weekly training volume, hours/week	5.8 ± 2.5	5.6 ± 2.6	6.1 ± 2.4
Sport initiation before 12 years of age	39 (27.7%)	23 (26.4%)	16 (29.6%)
Participation in multiple sports	35 (24.8%)	22 (25.3%)	13 (24.1%)
Sport category			
Endurance sports	32 (22.7%)	19 (21.8%)	13 (24.1%)
Power sports	20 (14.2%)	12 (13.8%)	8 (14.8%)
Skill/dexterity sports	30 (21.3%)	18 (20.7%)	12 (22.2%)
Mixed sports	59 (41.8%)	38 (43.7%)	21 (38.9%)

**Table 2:** Reasons for referral to specialist PM&R evaluation among referred athletes (*n* = 54).

Referral Category	Clinical Suspicion	*n*
Suspected scoliosis (hump deformity)	Trunk asymmetry on forward bending test	22
Foot alignment disorders	Flatfoot with hindfoot valgus	12
	Cavus foot	6
Thoracic hyperkyphosis with dorsal pain	Postural hyperkyphosis	5
Lower limb length discrepancy	Clinical asymmetry	4
Other musculoskeletal complaints	Various conditions	7

Detailed clinical features of the “other” category included dorsal pain in flat back posture, chronic low back pain, lumbar pain with sacral tenderness, bilateral hallux valgus, and heel pain. Referral indications were not mutually exclusive; individual athletes could contribute to more than one category. Accordingly, the total number of referral indications (*n* = 56) exceeded the number of referred athletes (*n* = 54).

**Table 3:** Diagnostic outcomes, imaging investigations and management among athletes completing PM&R evaluation (*n* = 40).

Referral Category	Imaging Performed	Final Diagnosis	Management
Suspected scoliosis (*n* = 16)	Spine X-ray (16), MRI (1)	Congenital scoliosis (1), idiopathic scoliosis (15)	Surgery (1), brace + therapeutic exercises (5), therapeutic exercises (6), follow-up (4)
Flatfoot with hindfoot valgus (*n* = 9)	X-ray (2)	Flexible flatfoot	Plantar orthoses (9)
Cavus foot (*n* = 5)	X-ray (1)	Cavus foot deformity	Orthoses + exercises (3), exercises (2)
Thoracic hyperkyphosis (*n* = 3)	X-ray (2)	Postural hyperkyphosis	Corrective exercises + follow-up (3), brace (1)
Limb length discrepancy (*n* = 3)	Clinical evaluation	Significant length discrepancy	Unilateral plantar lift (3)
Other conditions (*n* = 5)	X-ray (2), MRI (2)	Sever disease (1), flat back syndrome (1), lumbar disk protrusion (1), spondylolysis with anterolisthesis (1), bilateral hallux valgus (1)	Conservative management/orthoses/rehabilitation

## Data Availability

The data supporting the findings of this study are available from the corresponding author upon reasonable request.
